# Color-Matching and Blending-Effect of Universal Shade Bulk-Fill-Resin-Composite in Resin-Composite-Models and Natural Teeth

**DOI:** 10.1155/2016/4183432

**Published:** 2016-10-23

**Authors:** Rasha M. Abdelraouf, Nour A. Habib

**Affiliations:** Faculty of Oral and Dental Medicine, Cairo University, Cairo, Egypt

## Abstract

*Objectives*. To assess visually color-matching and blending-effect (BE) of a universal shade bulk-fill-resin-composite placed in resin-composite-models with different shades and cavity sizes and in natural teeth (extracted and patients' teeth).* Materials and Methods*. Resin-composite-discs (10 mm × 1 mm) were prepared of universal shade composite and resin-composite of shades: A1, A2, A3, A3.5, and A4. Spectrophotometric-color-measurement was performed to calculate color-difference (Δ*E*) between the universal shade and shaded-resin-composites discs and determine their translucency-parameter (TP). Visual assessment was performed by seven normal-color-vision-observers to determine the color-matching between the universal shade and each shade, under Illuminant D65. Color-matching visual scoring (VS) values were expressed numerically (1–5): 1: mismatch/totally unacceptable, 2: Poor-Match/hardly acceptable, 3: Good-Match/acceptable, 4: Close-Match/small-difference, and 5: Exact-Match/no-color-difference. Occlusal cavities of different sizes were prepared in teeth-like resin-composite-models with shades A1, A2, A3, A3.5, and A4. The cavities were filled by the universal shade composite. The same scale was used to score color-matching between the fillings and composite-models. BE was calculated as difference in mean-visual-scores in models and that of discs. Extracted teeth with two different class I-cavity sizes as well as ten patients' lower posterior molars with occlusal caries were prepared, filled by universal shade composite, and assessed similarly.* Results*. In models, the universal shade composite showed close matching in the different cavity sizes and surrounding shades (4 ≤ VS < 5) (BE = 0.6–2.9 in small cavities and 0.5–2.8 in large cavities). In extracted teeth, there was good-to-close color-matching (VS = 3.7–4.4 in small cavities, BE = 2.5–3.2) (VS = 3–3.5, BE = 1.8–2.3 in large cavities). In patients' molars, the universal shade composite showed good-matching (VS = 3–3.3, BE = −0.9–2.1).* Conclusions*. Color-matching of universal shade resin-composite was satisfactory rather than perfect in patients' teeth.

## 1. Introduction

Blending-effect (BE) of a restorative dental material refers to its ability to acquire a color resembling that of the adjacent structure [1]. Some manufacturers describe their dental products with this blending potential by having a chameleon effect [2].

A main previous research by Paravina et al. [3] investigated the blending-effect of shaded resin-composites related to restoration size. The authors used two main specimens' designs; one was composite ring (shade C2) with a central hole (2, 4, or 6 mm) filled with resin-composite (shades A2 and B2 of two different brands). This combined specimens design resembled teeth cavity walls filled by restorations. The other design was single composite discs from the same materials. Visual assessments were performed using a scale from 1 to 5, where score “1” was total mismatch while score “5” was Exact-Match. Visual scoring (VS) was performed twice; the first assessment was performed for the degree of color-matching between two single resin-composite separate discs: the surrounding shade composite discs (shade C2) versus the restoration shade discs (either shade A2 or B2). Meanwhile, the other assessment was performed for the combined specimens, where VS was performed for the filling (shades A2 and B2) within the composite ring (shade C2) with the different cavity sizes. BE was calculated as the difference in scores between the VS of the combined specimens and the VS of the single composite specimens (separate discs of restoration and surrounding shades). In addition, the single composite discs were evaluated by a spectrophotometer to determine their color parameter and TP. It was concluded that the BE of the fillings increased with the decrease of restoration size and the increase of the filling material translucency. It was also found that BE increased when the color-difference between the restoration and the surrounding walls decreased [3].

The blending-effect of layered composite was evaluated in another study by the same authors [1]. The previous methodology was followed but the combined specimens had a base simulating the pulpal floor, not just a ring. It was found that BE was composite and shade dependent [1].

Universal shade bulk-fill composite, in addition to its increased curing depth, is claimed by the manufacturer to have chameleon or BE. There is no published research on the blending capacity of this universal shade composite. The purpose of this study was to assess the color-matching and the BE of the universal shade composite in different restoration sizes and surrounding shades in resin-composite-models and natural teeth (extracted and patients' teeth).

## 2. Materials and Methods

**Figure 1:**
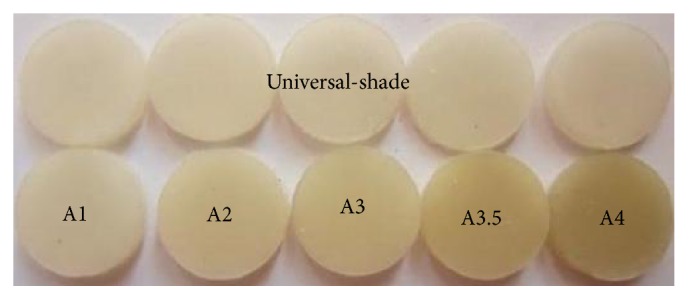
Resin-composite-discs.

**Figure 2:**
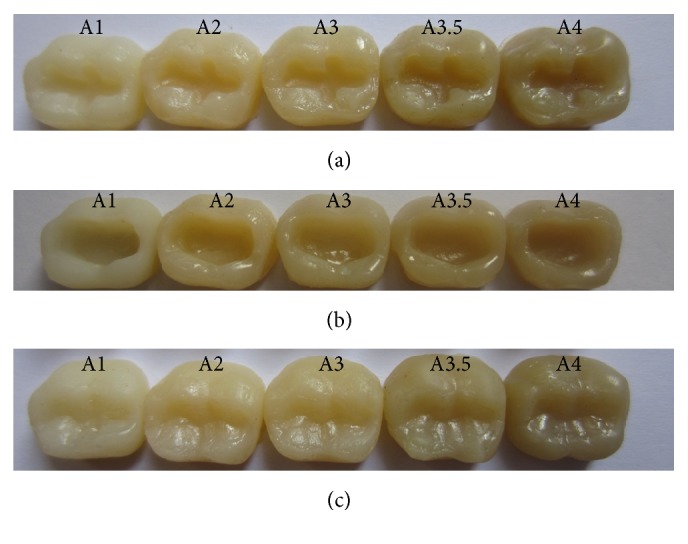
Teeth-like resin-composite-models shades A1, A2, A3, A3.5, and A4. (a) Small cavity preparation. (b) Larger cavity preparation. (c) After being filled with universal shade bulk-fill resin-composite.

The following materials were used in this study: universal shade bulk-fill resin-composite and five composite shades (A1, A2, A3, A3.5, and A4) (Table 1). The previous shaded composites were used for preparing discs (Figure 1) as well as teeth-like models (Figures 2(a) and 2(b)). Meanwhile the universal shade composites were used as discs and as a filling material for these models (Figure 2(c)), extracted teeth (Figure 5) and patients' teeth (Figures 6 [Fig fig7]–8).

### 2.1. Discs Preparation and Instrumental and Visual Color Assessment

Thirty-six discs (10 mm × 1 mm) were prepared from the universal shade and shaded resin-composites (*n* = 6/shade), Figure 1. To obtain the discs, the resin-composite was placed in a Teflon mold, flattened by glass slab and light-cured for 60 seconds using a light-curing-unit (Mini LED, Satelec, Acteon, France).

The color parameters of the discs were measured using a spectrophotometer (UV- Shimadzu 3101 PC-Spectrophotometer, Tokyo, Japan). The 10° observer function was used with the CIE Illuminant D65. Δ*E* between the universal shade and shaded-resin-composites discs were calculated according to the following equation: Δ*E* = [(Δ*L* ^*∗*^)^2^ + (Δ*a* ^*∗*^)^2^ + (Δ*b* ^*∗*^)^2^]^1/2^, where *L* ^*∗*^, *a* ^*∗*^, and *b* ^*∗*^ are the CIELAB color coordinates [4].

The TP of the discs was calculated as the color-difference between specimens placed against white and black backgrounds [5, 6]. A high TP value indicates high translucency [7]. TP = [(*L*_*W*_^*∗*^ − *L*_*B*_^*∗*^)^2^ + (*a*_*W*_^*∗*^ − *a*_*B*_^*∗*^)^2^ + (*b*_*W*_^*∗*^ − *b*_*B*_^*∗*^)^2^]^1/2^, where *L* ^*∗*^, *a* ^*∗*^, and *b* ^*∗*^ are the CIELAB color coordinates, while *W* and *B* refer to the values for each specimen against the white and black backgrounds, respectively [8]. The black standard tile (*L* ^*∗*^ = 11.24, *a* ^*∗*^ = 0.17, and *b* ^*∗*^ = 0.28) and the white standard tile (*L* ^*∗*^ = 95.72, *a* ^*∗*^ = 0.60, and *b* ^*∗*^ = 3.54) were used as the standard backgrounds for the specimens during the color measurements. The results of Δ*E* and TP were analyzed by one-way ANOVA followed by Bonferroni's post hoc test to detect significant differences in values (*P* ≤ 0.05). Statistical analysis was performed with IBM® SPSS® Statistics Version 20 for Windows.

Visual assessment was performed by seven female observers (four dentists and three scientists), with normal-color-vision as checked by Ishiara test. To determine the color-matching between the universal shade and each shaded composite disc, color-matching visual scoring (VS) values were expressed numerically as follows: 1: mismatch/totally unacceptable, 2: Poor-Match/hardly acceptable, 3: Good-Match/acceptable, 4: Close-Match/small-difference, and 5: Exact-Match/no-color-difference. All observers were trained in color-matching and taught about the used grading system before the assessment.

A color assessment cabinet (VeriVide CAC60, England), with neutral grey walls and floor, was used for the visual evaluation. Illuminant D65 was selected. The cabinet had an oblique grey specimens' holder providing a 45° angle between the specimens and the illuminant. The observers placed their head on a head holder allowing an observation distance of 25 cm from the specimens and looking perpendicularly upon them. Thus, the illuminant/viewing geometry was 45°/normal. During assessments, external lights were turned off and, after an adaptation period, the observers scored the color-matching between the universal shade and shaded composite discs which were in edge contact. Results were recorded and mean values were calculated. Inter- and intraobserver agreements were also recorded.

The interobserver agreement was calculated as the mean value of the highest percentage of observers that graded specimens of each shade identically (i.e., between different observers). The intraobserver agreement was calculated as the mean value of the highest percentage of identical scores for the six specimens of the same shade by a specific observer (i.e., for each observer).

### 2.2. Resin-Composite-Models Construction and Visual Assessment

**Figure 3:**
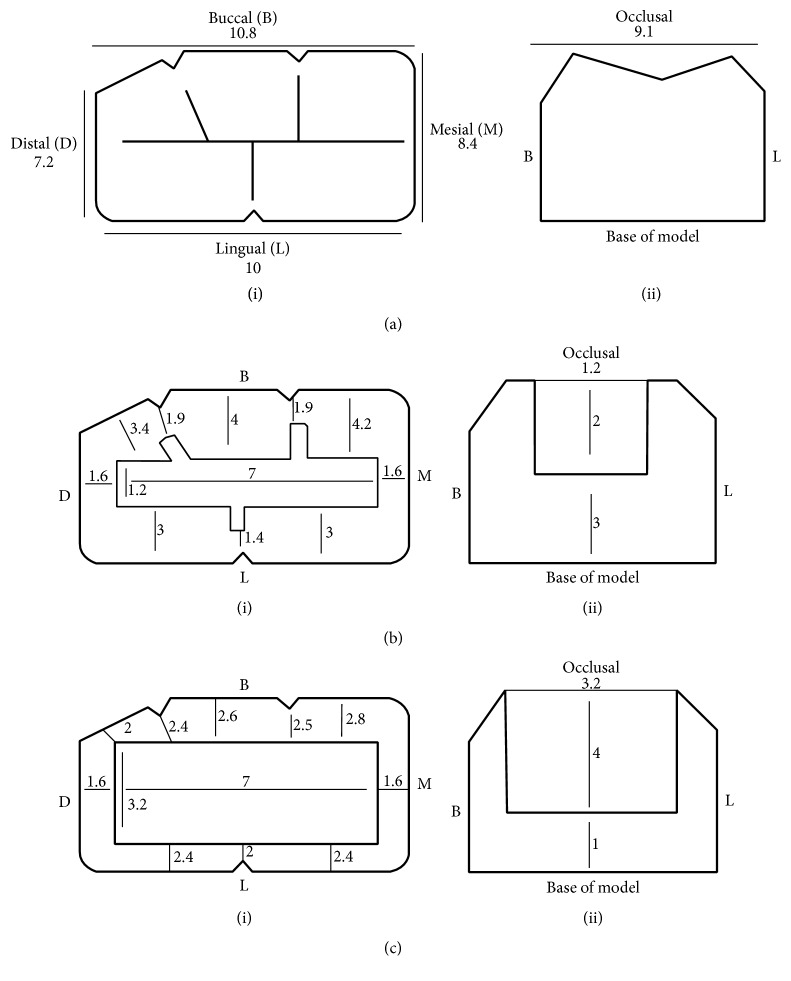
Diagram representing dimensions of the models in millimetres (mm). (a) Tooth without cavity preparation. (b) Small cavity preparation. (c) Larger cavity preparation ((i) top view, (ii) lateral view).

**Figure 4:**
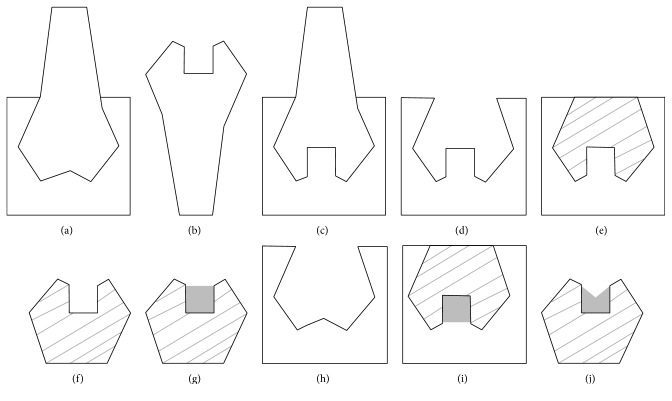
Steps of composite-models fabrication: (a) taking rubber index. (b) Prepared cavity in acrylic tooth. (c) Impression for acrylic tooth with cavity. (d) Rubber impression used as mold for models fabrication. (e) Resin-composite in the rubber mold. (f) Resin-composite-model. (g) Uncured universal shade composite filling in model. (h) Rubber index for intact acrylic tooth obtained from first step. (i) Insertion of model with filling in the index. (j) Filling with standardized occlusal anatomy.

The dimensions of the models and the prepared cavities are shown in Figure 3, while the steps of models fabrication are represented diagrammatically in Figure 4. Two acrylic lower first molars (Elbanna, Cairo, Egypt) were used for construction of the different resin-composite shade models (surface width: buccal (B) width = 10.8 mm, lingual (L) width = 10 mm, mesial (M) width = 8.4 mm, and distal (D) width = 7.2 mm), Figure 3(a). Rubber impressions for the occlusal anatomy were taken (Speedex Putty, Coltene/Whaledent, Altstätten, Switzerland), Figure 4(a). These impressions were used as indices to give an identical surface anatomy of the universal shade bulk-fill resin-composite restorations.

Class I occlusal cavities were prepared in each acrylic tooth, one with a small cavity size (dimensions: BL = 1.2 mm, MD = 7 mm, and depth = 2 mm), Figure 3(b), while the other is with a larger cavity size (dimensions: BL = 3.2 mm, MD = 7 mm, and depth = 4 mm), Figure 3(c). The thicknesses of the resin-composite from the bottom of the cavities to the base of the models were 3 mm for the small cavity and 1 mm for the larger cavity, Figures 3(b)(ii) and 3(c)(ii).

Rubber base impressions were taken for the coronal portions of the acrylic teeth after the cavities being prepared, Figure 4(c). These impressions were used to make resin-composite duplicates of various shades for the coronal portions with reproducible cavity dimensions.

To construct each resin-composite-model, the previous impression was filled with a shaded resin-composite, Figure 4(e), and a glass slab was placed on the filled index. The resin-composite was cured for 60 seconds. The resin-composites model was retrieved out of the impression, Figure 4(f), and an additional light curing was performed from the occlusal, buccal, lingual, mesial, and distal directions, 60 seconds each. The surface of the model was finished and polished to remove the oxygen inhibition layer (Politip P/F Ivoclar, Vivadent, Schaan, Liechtenstein).

The models with the identical prepared cavities were restored by the universal shade bulk-fill resin-composite, Figure 4(g), and then inserted into the impressions taken for the acrylic teeth before cavity preparation, Figures 4(h) and 4(i). Light curing was performed through the base of each model for 60 seconds. Then the model was removed from the index, Figure 4(j), and light-cured for an additional 60 seconds from the occlusal direction and finished and polished as before.

The color-matching VS for the fillings versus the different shades of models with different cavity sizes was assessed as before. BE was calculated as a difference in color-matching scores for the models versus that for composite disc of the same shade (BE = VS of models − VS of separate discs). The interobserver and intraobserver agreements were calculated as before.

### 2.3. Extracted Teeth and Visual Assessment

**Figure 5:**
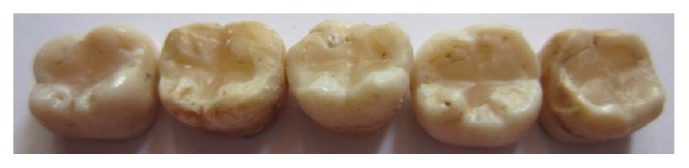
Natural teeth filled by universal shade composite.

Freshly extracted human lower molars sound natural teeth were selected for this study. The shades of the teeth were assessed by a spectrophotometer (VITA Easyshade, VITA Zahnfabrik, Germany). Two different class I-cavity sizes were prepared as before, filled by universal shade composite, Figure 5, and assessed similarly. The BE and interobserver and intraobserver agreements were calculated as before.

### 2.4. Patients' Teeth and Visual Assessment

**Figure 6:**
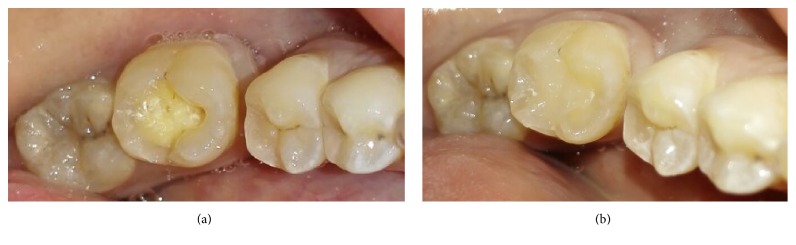
Case I: (a) prepared cavity in lower molar. (b) After being filled by universal shade composite.

**Figure 7 fig7:**
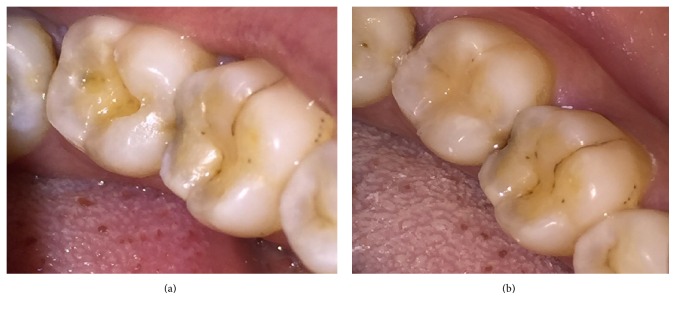
Case II: (a) prepared cavity in lower molar. (b) After being filled by universal shade composite.

**Figure 8:**
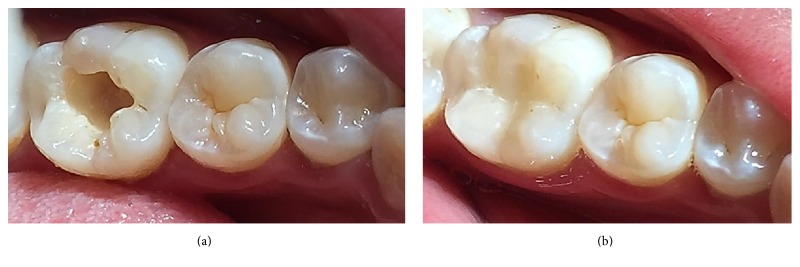
Case III: (a) prepared cavity in lower molar. (b) After being filled by universal shade composite.

Ten patients with occlusal caries in lower posterior molars were selected to participate in this study, after getting the approval of the research ethics committee and the patients' informed consents. The shades of their teeth were determined using VITA Easyshade spectrophotometer. After class I cavities had been prepared, a 37% phosphoric acid etching gel (Eco-Etch, Ivoclar Vivadent, Liechtenstein) was applied for 15 seconds, then rinsed, and air-dried. A bonding agent (Universal Single Bond, 3M ESPE, Germany) was applied to the teeth in a rubbing motion for 20 seconds. Then, a gentle stream of air was applied over the bonding agent for 5 seconds and then it was light-cured for 10 seconds. The universal shade bulk-fill composites (X-Tra Fil, Voco, Cuxhaven, Germany) were used to fill the cavities and then light-cured for 20 seconds. Photographs of some representative clinical cases are shown in Figures 6 [Fig fig7]–8.

Visual assessment for the filling color-matching was performed by the same observers using the same scoring system. The assessment was performed in a clinic related to the dental school (105 m^2^ room with ceiling height of 2.82 m and eliminated with color corrected florescent lambs). The walls of the clinic were painted with an off-white color (*L* ^*∗*^ = 63.5, *a* ^*∗*^ = −2, and *b* ^*∗*^ = 6.8).

The patients were seated on a dental unit (A-dec 200, USA) in upright position. The light source of the dental unit (high intensity: 17,000 luxes and color temperature: 4800 Kelvins) was focused on the patients' mouth from the distance of one meter. Then, each patient was asked to open his mouth and the observers assessed the color-matching of the filled tooth. The observers performed the evaluation twice for the color-matching of the universal shade composite filling. The BE and interobserver and intraobserver agreements were calculated as before.

## 3. Results

Δ*E* values of the universal shade versus the shaded composite discs and their TP are presented in Table 2. Δ*E* increased as the shades become darker. The universal shade showed the highest TP (26.7 ± 0.7).

The VS values of the universal shade composite versus the shaded composite discs and models are shown in Table 3, in addition to the BE in the models. The VS of the separate discs showed close color match between universal shade and shade A1 composite (VS = 4.2), Good-Match in shades A2 and A3 (VS = 3.2 and 3, resp.), and mismatch in shades A3.5 and A4, respectively (VS = 1.4 and 1.2, resp.). On the other hand, the VS of the models showed there was close matching between the universal shade fillings and the surrounding composite in the different cavity sizes and surrounding shades (4 ≤ VS < 5).

Since BE is calculated by subtracting VS of separate discs from that in models, thus, BE values for shade A1 in small and large cavities were 0.6 (= 4.8 subtracted by 4.2) and 0.5 (= 4.7 subtracted by 4.2), respectively. Meanwhile BE of shade A4 were 2.9 (= 4.1 subtracted by 1.2) and 2.8 (= 4 subtracted by 1.2), respectively. The BE values in composite-models increased with darker shades and increasing the color-difference between the filling and surrounding shade separately.

The shades of the extracted teeth were A4 as measured by the VITA Easyshade spectrophotometer.  Table 4 shows the VS and BE of universal shade composite in extracted teeth, where BE is calculated by subtracting the VS of shade A4 discs (1.2) from the mean VS values of the teeth. The universal shade composite showed good-to-close matching in extracted teeth in small cavities (VS = 3.7–4.4). However, these values decrease in larger cavities (VS = 3–3.5). Thus, the color-matching and BE decrease with increase in cavity size.

Table 5 shows the shades, VS, and BE of universal shade resin-composite in the patients' teeth. Good color-matching was observed in vivo (VS = 3–3.3). Yet close matching was not selected by any observer. The BE ranged from −0.9 to 2.1 in darker shades.

The interobserver agreement was 82.2% for single composite discs and 75.9% and 82.2% for models with small and large cavities, respectively. Meanwhile interobserver agreements for extracted teeth with small and large cavities were 79.3% and 89.4%, respectively. In the patients' teeth, the interobserver agreement was 92.5%.

However, the intraobserver agreements for single composite discs and models with small and large cavities were 86.6%, 84.3%, and 87.3%, respectively. Meanwhile intraobserver agreements for extracted teeth with small and large cavities and the patients' teeth were 86%, 91.9%, and 93.2%, respectively.

## 4. Discussion

Universal shade bulk fill resin-composite may represent a smart solution in color-matching if it blends with the adjacent tooth structure shade, beside its increased curing depth. This material is indicated for posterior fillings (classes I and II), not in anterior teeth (classes III and IV). In the latter case, the high translucency of this resin-composite would transmit the background color of the oral cavity.

In this study, resin-composite-models were constructed to evaluate the BE, rather than disc-shaped specimens, in an attempt to resemble the natural teeth and restorations in morphology and dimensions. In addition, these models standardized the sizes of the cavities and restorations as well as the surrounding wall thicknesses and shades. However, discs were used for spectrophotometric measurements as they had flat surface for accurate results.

The BE in this study were determined by the difference in mean scores (mean category values) as it was shown previously in another research that the computation of mean category values was valid and that differences between mean category values can be used to represent the blending-effect [3].

The universal shade composite fillings showed close color-matching in the composite-models despite their great color variation (shades A1, A2, A3, A3.5, and A4), as verified by Δ*E* values. Although there are several formulas for color-difference calculation, the most commonly used one in dental research is obtained from the CIE-*L* ^*∗*^ *a* ^*∗*^ *b* ^*∗*^ system [4]. This approximates uniform distance among the color coordinates covering entirely the visual color space [9].

The high color-matching of the universal shade composite might be attributed to its high translucency reflecting the shade of the surrounding walls, even with different shades and translucency. This was in agreement with Paravina et al. [3] who concluded that the BE increased with increasing the translucency.

It was noticed that the BE of the universal shade composite in darker models was higher as numerical values, compared to that in lighter one. Yet, its color-matching was higher in lighter shades. In our study, the BE values increased with increasing the color-difference between the universal shade and the surrounding composites. This was contradictory to what was concluded by Paravina et al. [3] that the BE increased with decrease in color-difference. This might be attributed to the difference in methodology. In this prior study [3], the surrounding shade was constant, while the fillings were different. Contrarily, in our research, the universal shade composite is fixed and the surrounding shades were multiple. In the latter case, the BE, calculated as before, may give misleading values when comparing the blending of multiple shades, as the visual scores of the separate discs decreased with darker shades. The color-matching scores could be more representative.

Comparing the BE among the different cavity sizes within the same model shades, it was shown that the BE value decreased when the size of the cavity increased. This was in agreement with Paravina et al. [3] who concluded that the BE was inversely related to the restoration size.

It was stated by Paravina et al. [3] that “the blending effect is the opposite of simultaneous color contrast [10–13].” Simultaneous color contrast occurs when color shifts towards the complementary color of the surroundings [14]. On the contrary, BE occurs when colors seem to be closer when together than if they were viewed separately [3]. Contrast or blending occurrence depends on several factors among them the size [3, 15]. As in agreement with the previous studies, the decrease in restoration size may lead to an increase in BE, associated with decreasing the visual angle of subtense (2*θ*) calculated as follows: 2*θ* = arctan⁡(*r*/*d*), where *r* is the radius of a specimen and *d* is the distance from the observer [3, 16].

The BE of the extracted teeth differed from that of the models. This may be due to the difference in their nature; for example, natural teeth are multilayered and polychromatic [3]. The extracted teeth being nonvital with structural changes than vital ones, assessing the VS and BE of fillings in patients' mouth was a crucial step.

The inter- and intraobserver agreement in this study were relatively high as those observed in Paravina et al. [3] and contrary to the lower values reported by Barna et al. [17]. In the first study the interobserver agreement was 83% for single composite discs and 75% for combined specimens, while the intraobserver agreement was 88% for single discs and 81% for combined specimens [3]. Yet, in the latter, the interobserver agreement was 39%, while the intraobserver agreement was 22% [17]. This may be attributed to the difference in research methodology.

## 5. Conclusions

The universal shade bulk-fill resin-composite showed acceptable color-matching in vivo; however it may not be the optimal selection when esthetics is the patient's prime concern. It may be better to assess color-matching and blending-effect in vivo rather than in vitro as it is a better simulation of clinical condition.

## Figures and Tables

**Table 1:** Product, manufacturer, composite type, filler content, matrix composition, shade, and lot of used resin-composites.

Product	Manufacturer	Composite type	Filler content		Matrix composition	Shade	Lot
			% wt	% vol			
X-Tra Fil	Voco, Cuxhaven, Germany	Hybrid	86%	70.1%	Bis-GMA^*∗*^, UDMA^*∗*^, TEGDMA^*∗*^	Universal shade	1306188

Grandio	Voco, Cuxhaven, Germany	Nanohybrid	87%	71.4%	Bis-GMA^*∗*^, TEGDMA^*∗*^	A1	1305344
						A2	1307394
						A3	1308483
						A3.5	1232524
						A4	1305345

^*∗*^Bis-GMA: bisphenol A diglycidyl ether dimethacrylate; UDMA: urethane dimethacrylate; and TEGDMA: triethylene glycol dimethacrylate.

**Table 2:** Δ*E* values of universal-shade composite versus different shades composite discs and their TP.

	Universal-shade	A1	A2	A3	A3.5	A4	*P* value
Δ*E*	—	4.8 ± 0.9^a^	10.4 ± 0.5^b^	12.2 ± 0.4^c^	18 ± 0.4^d^	20.8 ± 0.9^e^	<0.001^*∗*^
TP	26.7 ± 0.7^a^	23.6 ± 0.4^b^	19.6 ± 0.6^c^	18.1 ± 0.5^d^	17.6 ± 0.3^e^	16.3 ± 0.3^f^	<0.001^*∗*^

*∗*: significant at *P* ≤ 0.05. Different superscripts are statistically significantly different per each row.

**Table 3:** The VS and BE values of universal-shade composite in different shades composite discs and models.

	A1	A2	A3	A3.5	A4
Discs	VS = 4.2 ± 0.5	VS = 3.2 ± 0.6	VS = 3 ± 0.6	VS = 1.4 ± 0.5	VS = 1.2 ± 0.4

Models: small cavity	VS = 4.8 ± 0.4	VS = 4.7 ± 0.5	VS = 4.6 ± 0.7	VS = 4.2 ± 0.9	VS = 4.1 ± 0.9
	BE = 0.6	BE = 1.5	BE = 1.6	BE = 2.8	BE = 2.9

Models: large cavity	VS = 4.7 ± 0.5	VS = 4.5 ± 0.4	VS = 4.2 ± 0.6	VS = 4.1 ± 0.8	VS = 4 ± 0.8
	BE = 0.5	BE = 1.3	BE = 1.2	BE = 2.7	BE = 2.8

**Table 4:** The VS and BE values of universal-shade composite in extracted teeth (shade A4).

Natural teeth: small cavity	VS = 3.7 ± 0.6	VS = 3.9 ± 0.8	VS = 4.1 ± 0.8	VS = 4.2 ± 0.7	VS = 4.4 ± 0.6
	BE = 2.5	BE = 2.7	BE = 2.9	BE = 3	BE = 3.2

Natural teeth: large-cavity	VS = 3 ± 0.9	VS = 3.1 ± 0.8	VS = 3.2 ± 0.6	VS = 3.3 ± 0.6	VS = 3.5 ± 0.8
	BE = 1.8	BE = 1.9	BE = 2	BE = 2.1	BE = 2.3

**Table 5:** The VS and BE values of universal-shade composite in patients' teeth.

Patient	1st	2nd	3rd	4th	5th	6th	7th	8th	9th	10th
Shade	A1	A2	A2	A3	A3	A3	A3.5	A3.5	A4	A4
VS	3.3 ± 0.3	3.1 ± 0.2	3.1 ± 0.3	3.2 ± 0.1	3.1 ± 0.3	3 ± 0.2	3.2 ± 0.2	3.2 ± 0.3	3 ± 0.1	3.3 ± 0.1
BE	−0.9	−0.1	−0.1	0.2	0.1	0	1.8	1.8	1.8	2.1
